# Bone Mineral Density Screening System Using CMOS-Sensor X-ray Detector

**DOI:** 10.3390/s21217148

**Published:** 2021-10-28

**Authors:** Areerat Maneerat, Sarinporn Visitsattapongse, Chuchart Pintavirooj

**Affiliations:** School of Engineering, King Mongkut’s Institute of Technology Ladkrabang, Bangkok 10520, Thailand; 60011210@kmitl.ac.th (A.M.); sarinporn.vi@kmitl.ac.th (S.V.)

**Keywords:** bone mineral density, dual energy X-ray, bone mineral content, DEXA

## Abstract

This research concerns a design and construction of a bone mineral density (BMD) and bone mineral content (BMC) measurement system based on dual energy X-ray absorptiometry (DEXA). An indirect X-ray detector is designed by optical coupling CMOS sensor with image on the intensifying screen. A dedicated microcontroller X-ray apparatus is used as an X-ray source to capture two energy level X-ray of middle phalanges bone of middle finger. The captured image is processed based on modified Beer-Lambert law to compute bone mineral density. Bone mineral content is also computed by determining the area of the phalanges bone using active contour. The designed bone mineral density (BMD) and bone mineral content (BMC) measurement system is low-cost and hence can be distributed at district hospital for screening purposes of Osteoporosis of the elderly. Compared with BMD measured from commercial model, BMD measurement of our system acquires linear relation with R2 equals 0.969. The mean square error between the normalized BMD value and that of the commercial model is 0.0000981.

## 1. Introduction

Bone is the basic unit of the human skeletal system that provides the framework and bears the weight of the body, protects the vital organs and supports mechanical movement of human body. Bone consists of the protein collagen which is the fiber link with a calcium phosphate salt, a substance that makes bones strong. Calcium salts in bone is accounted for 99 percent with 1 percent is in the blood. Osteoporosis is a condition that causes weakening of the bones because the mineral calcium in the bones decreases. Osteoporosis is a cause of humpback and bone fragility. Osteoporosis is called the “deadly silence” because the symptoms of the disease will move forward all the time without warning before. The patient will know when the bone has broken already. Osteoporosis was found in women than men because women have bone mass less than men. When women are menopausal and hormone estrogen decrease, which helps calcium to hold the bone down.

Diagnosis of osteoporosis in general requires bone mineral density (BMD) and bone mineral content (BMC) measurement. There exist three different well-known modalities that can be used for bone mineral density and content measurement including X-ray, ultrasonic and microwave modality. X-ray modality based on absorptiometry is the most widely used modality that has captured researcher attention during the last decade. Ligesh et al. [1] estimated bone mineral density using radiographic absorptiometry by taking a step-wedge bone phantom with digital X-ray. The measured gray-level values of all the steps as well as the corresponding known BMD values were used to create the calibration curve and later used for BMD estimation of the unknown gray level of the bone area. The disadvantage of the proposed technics of Ligesh et al. is that the technic is bone thickness and X-ray exposure parameter-setting dependent as only single energy X-ray is used. The calibration curve is then must be computed for each subject. Lee et al. [2] estimated bone mineral density by analyzing trabecular pattern on the distal radial bone. They defined trabecular pattern parameter (TTP) in which ROI of distal radial image is divided into many sub-blocs. The gray level in each sub-bloc is then normalized to scale 0 to 1. An averaged intensity in each sub-bloc is computed. TTP is then defined by the average of the averaged intensity of all sub-bloc. R^2^ of 0.92 is claimed for the linear relation between TTP and BMD. The proposed method of Lee et al. also suffered from bone thickness dependent and X-ray exposure parameter setting dependent. To solve the drawbacks of thickness dependent and X-ray exposure parameter setting dependent, modified Beer-Lambert law using two energy levels of X-ray are used to derive bone-mineral density [3]. The technics is called dual-energy X-ray absorptiometry or DEXA. In general, to acquire two energy levels, a high and low source tube voltages is alternately supplied to X-ray generator. Dendere et al. [4] proposed the system that includes an additional detector above the existing one in a slot-scanning radiography system to capture dual-energy images. The system successfully measured bone mineral density (BMD) of the middle phalanx of the middle finger with small error when compared with those from a commercial DEXA machine. Chiang et al. [5] used cone beam computed tomography (CBCT) to acquire the gray level distribution of trabecular jawbone of patients in different ages and determines the threshold of loose bone mineral density. The technic is used as screening propose, i.e., if the average gray value of patient’s CBCT image is less than some threshold, the dentist may need to examine the patient with DEXA. Even though bone mineral measurement based on dual-energy X-ray absorptiometry is the gold standard recommended by World Health Organization, it only provides a 2-D projected measurement of the bone mineral density. For improved accuracy of bone mineral measurement, clinical computed tomography (CT) imaging can be used for measurement of volumetric bone mineral density. 3D bone mineral density measurement uses a series of K2HPO4 solution phantoms with various concentrations as a reference standard [6]. The equivalent volumetric BMDs of the solution phantoms can be calculated by their known percent weights of K2HPO4. Regression analysis is then used to map the CT number with the volumetric BMD. Even though computed tomography imaging can provide more accurate estimation for bone mineral density, the technics suffered from extra radiation dosage delivered to the patient. More research on bone mineral density based on X-ray modalities can be found in [7,8,9,10].

Quantitative ultrasound (QUS) has become increasingly potential methods for measuring bone mineral density because of its advantages of low-cost, portable, easy to access, non-radiation-based modality and high precision. Moreover, as ultrasonic is mechanical wave, its propagation through the bone is affected by its physical structure especially bone composition and bone mass and hence can be used to assess bone. Normally, the investigations are performed on the heel bone of ulna bone as it mainly composed of a trabecular bone, which is easy to access. Lei et al. [11] developed a high-speed synchronous acquisition of ultrasonic waves to acquire broadband ultrasonic attenuation (BUA) and speed of sound (SOS) and used them to diagnose and forecast the bone mass loss, osteoporosis. Samir et al. [12] developed a prototype of ultrasound forearm bone densitometer to measure BMD. The prototype of the device was constructed using a pair of wideband, flat, composite ultrasonic transducers operating at a central frequency of 0.5 MHz. Ultrasonic transmit time and ultrasonic attenuation are used as the key parameters for bone mineral density estimation. The proposed technic demonstrated high sensitivity to bone mass as compared with peripheral DEXA bone densitometer and high accuracy to estimate the BMD at ulna bone region. Kaufman et al. [13] developed a quantitative ultrasound device that measures two time-delay parameters. The first time-delay parameter is defined as the difference between the transit time of an ultrasound pulse to travel through soft-tissue, cortex and medullary cavity, and the transit time through soft tissue only of equal overall distance; where the second time-delay parameter is defined as the difference between the transit time of an ultrasound pulse to travel through soft-tissue and cortex only, and the transit time through soft tissue only again of equal overall distance. The square root of the product of these two time-delay parameters is then used to estimate bone mineral density of the radial bone. Validated with clinical DEXA UltraScan 650 model, the plot between the BMD measured from DEXA and the square root of the product of these two time-delay parameters demonstrates a linear relation with *R^2^* of 0.92. More research on QUS for bone density measurement can referred to [14,15,16]. 

Microwave modality is an alternative promising technic that can be used for bone mineral density assessment. Microwave is an electromagnetic wave of which the energy is between ultrasound and X-ray. As microwave is a non-ionization radiation, it is hence healthier than X-ray and yet more penetrable than the ultrasound. The microwave modality has gain increasing attention recently due to the development of receiving microwave antenna and microwave source technology. Augustine et al. [17] developed an ultra-wideband (UWB) measurement system for bone mineral evaluation. UWB frequencies from 1–8 GHz has been used and received reflected signal from the bone phantom. The resonant frequencies of the UWB Vivaldi antenna used in the research are highly dependent on dielectric properties of bone and hence can be used to evaluate bone mineral density. Kerketta and Ghosh [18] developed a monopole UVB microwave antenna ranging from 3 GHz to 10 GHz to study on a level of bone mineral content. A change in relative attenuation of the microwave signal when passed through the bone is related bone mineral density. The experiment is conducted on animal bone phantom with various mass density. Cruz et al. [19] designed a 2.4 GHz antenna to measure the relative attenuation of the transmitted microwave signal traversing through several samples of bovine bone, silica, and bone powder. Support vector machine is further applied to classify a sample as having low or high bone density. Barros et al. [20] designed the 2.4 GHz microwave antenna to detect the levels of attenuation of the transmitted microwave through a phantom of various bone density. Wavelet Transform was applied to the receiving electromagnetic signals, and it can be used to extract relevant features of these signals for bone mineral classification. Even though microwave has gained increasing momentum for bone mineral assessment, the research is mostly conducted in the bone phantom. Furthermore, under current microwave antenna technology, the antenna with smaller size still cannot be developed. The limitation hampers the development of microwave imaging that can be used in in-vivo clinical application. 

In our research, we developed a bone mineral density measurement system based on dual energy X-ray. The salient aspects and/or contributions of this paper are enumerated as follows.

(1) We develop a dedicated microcontroller digital X-ray apparatus that can be interfaced with personal computer to control the bone mineral density measurement process.
(2) We design a low-cost upgradable indirect X-ray detector by using CMOS sensor mounted in a light-protective case to capture image on the X-ray phosphor intensify screen. By concatenating multiple CMOS sensors, resolution of our X-ray digital system can be increased comparable the commercial X-ray detector panel.
(3) When compared the commercial, our bone mineral measurement system is low cost and can be used as screening examination of bone mineral deficiency at district hospital.
(4) We optimized the setting X-ray exposure parameters such that the proposed bone mineral density measurement system based on dual energy X-ray delivers low dose to the subject. The effective dose is as low as that of the commercial.
(5) The portable bone mineral density measurement system based on dual energy X-ray is re-designed and constructed with the same concept proposed in this research.

## 2. Materials and Methods

The proposed bone density measurement is based on using dual-energy X-ray. We developed a dedicated microprocessor-controlled 50-mA 100-kVp stationary-anode X-ray apparatus that is capable of being controlled by personal computer via serial communication. The two-dimension X-ray detector is based on an indirect X-ray detector in which CMOS sensor [21] is installed in light-proof lead-lining case to capture image on intensifying screen that are radiated by X-ray. The captured image from CMOS sensor is transferred to personal computer for further processing with inhouse software using OpenCV image processing library [22]. 

### 2.1. Indirect X-ray Detection Design

This section concerns a design and construction of a low-cost indirect X-ray detector panel by using CMOS sensors coupled with optical lens to capture image of an intensifying screen. The Micron^®^ Imaging MT9MOOl CMOS sensor used in this research is an SXGA-format with a 1/2-inch CMOS active-pixel digital image sensor. The resolution is of 1280 H × 1024 V. It incorporates sophisticated camera functions on-chip such as windowing, column and row skip mode, and snapshot mode. It is programmable via a simple two-wire serial interface. This mega pixel CMOS image sensor features Digital-Clarity Micron’s breakthrough low-noise CMOS imaging technology that achieves CMOS image quality (based on signal-to-noise ratio and low-light sensitivity) while maintaining the inherent size, cost, and integration advantages of CMOS. Table 1 shows the key parameter of CMOS sensor.

**Figure 1:**
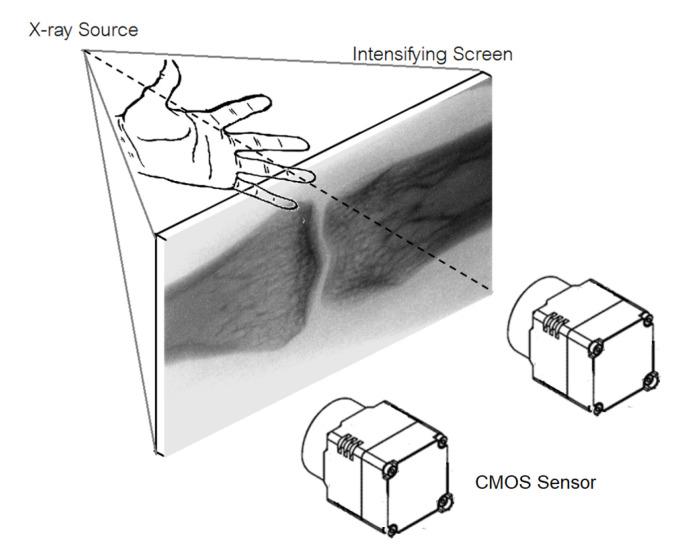
CMOS-sensor X-ray Detector.

One of the key factors affecting the performance of the proposed indirect X-ray is the scintillator. The most used scintillator is Gadolinium based phosphor—the so-called GADOX, Cadmium Tungstate, CdWO4, and Thallium doped Cesium Iodine, CsT (TI). [23] The new scintillation material includes Gadolinium based ceramic scintillator, such as Gd_2_O_2_S_2_ and Y_2_O_3_Gd_2_O_3_ (YGO), which are used in advanced medial CT detector. The suitability of specific application of scintillator depends upon the following characteristics: (1) absorption coefficient, (2) afterglow and (3) light output. The absorption coefficient and the afterglow of Gadolinium based ceramic scintillator is distinguished when compared with Cadmium Tungstate and Thallium doped Cesium Iodine. Yet Cesium Iodine yields the prominent light output. To coupling scintillator with any photo detector, the spectral characteristics of the light output need to be considered, i.e., the wavelength of the emitted light of the scintillator should match with response of the CMOS Sensor. This research GADOX intensifying screen is used. The design of our low-cost indirect X-ray detector system is shown in Figure 1. The system consisted of one or multiple of CMOS sensors installed in the back plate of detector light-proof box. The front plate of detector light-proof box is attached with Gadox (Gd2O2S: Tb) high-sensitive intensifying screen. When X-ray traversed through the object and exposed to the intensifying screen, the intensifying screen will convert incident X-ray energy to visible light. The intensity of light at any particular location depends on the intensity of incident X-ray which in turn depends on the attenuation coefficient of the object radiated by X-ray. To increase field of view, multiple CMOS Sensors can be used to capture image appeared on the intensifying screen. In such case, image processing is further required to stich the image obtained from each CMOS Sensor. The overall digital image processing used in X-ray detection can be divided into 4 step including (i) distortion correction, (ii) noise reduction, (iv) image registration and (iv) image stitching 

#### 2.1.1. Distortion Correction

Our indirect X-ray detector uses CMOS sensor capturing image on intensifying screen. Distance between CMOS sensor and intensifying screen depends on the focal length of the coupled optical lens on the CMOS sensor. Short focal-length coupling lens is preferable to the long focal-length coupling lens so the system will be more compact. Using short focal length coupling lens, however, there will be a distortion of the capture image; the so-called pincushion or barrel distortion [24]. To correct distortion, polynomial warping is used. The chess box phantom is captured with the CMOS sensor. The chess-board corner points of the captured image are extracted. Polynomial warping is then applied to non-linearly transform the distorted corner points to the original perfect-square non-distorted points. The quadratic polynomial transformation is defined as,

Y=T⋅X

or

(1) $$\left[\begin{array}{c} u\\ v\\ 1 \end{array}\right]=\left[\begin{array}{cccccc} a_{5} & a_{4} & a_{3} & a_{2} & a_{1} & a_{0}\\ b_{5} & b_{4} & b_{3} & b_{2} & b_{1} & b_{0} \end{array}\right]\left[\begin{array}{c} x^{2}\\ y^{2}\\ xy\\ x\\ y\\ 1 \end{array}\right]$$

where Y is the distorted points and X is the original non-distorted points. For a data of *n* points,

$$Y=\left[\begin{array}{cccc} u_{1} & u_{2} & \cdots & u_{n}\\ v_{1} & v_{2} & \cdots & v_{n}\\ 1 & 1 & \cdots & 1 \end{array}\right]$$

$$X=\left[\begin{array}{cccc} x_{1}^{2} & x_{2}^{2} & \cdots & x_{n}^{2}\\ y_{1}^{2} & y_{2}^{2} & \cdots & y_{n}^{2}\\ x_{1}y_{1} & x_{2}y_{2} & \cdots & x_{n}y_{n}\\ x_{1} & x_{2} & \cdots & x_{n}\\ y_{1} & y_{2} & \cdots & y_{n}\\ 1 & 1 & \ldots & 1 \end{array}\right]$$

In addition,

$$T=\left[\begin{array}{cccccc} a_{5} & a_{4} & a_{3} & a_{2} & a_{1} & a_{0}\\ b_{5} & b_{4} & b_{3} & b_{2} & b_{1} & b_{0} \end{array}\right]$$

The transformation matrix T can be recovered from at least six pairs of corresponding points and estimated using least square error (LSE). The LSE solution of T is given as,

(2) $$T={\left(X^{T}X\right)}^{-1}(X^{T}Y)$$

**Figure 2:**
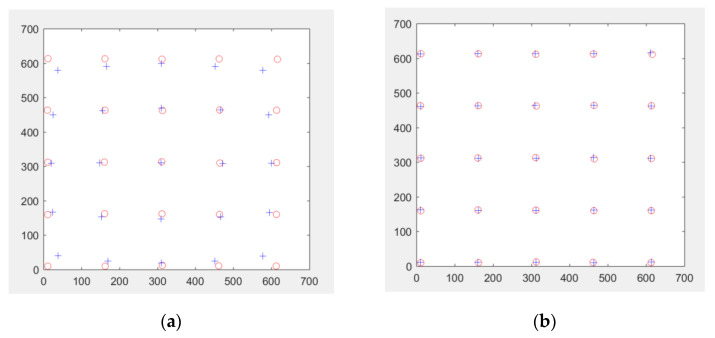
Distortion correction; (**a**) before correction, (**b**) after correction. Square is the non-distorted point of chess board phantom and + is the distorted capture point of the chess board phantom.

The estimated transformation T is then used to correction the distortion as seen in Figure 2. 

#### 2.1.2. Noise Reduction

**Figure 3:**
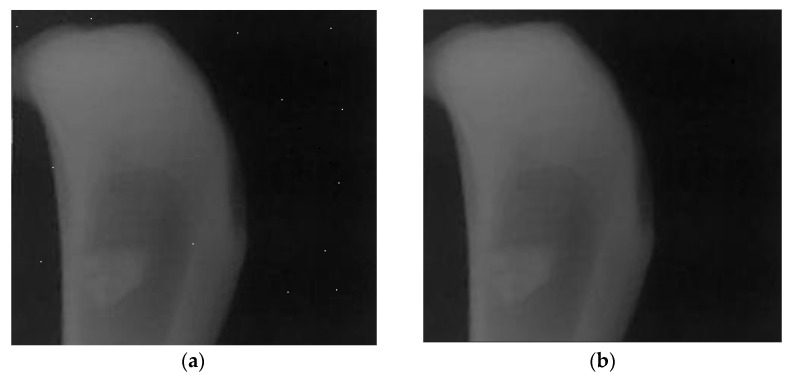
Salt and pepper noise reduction using a median filtering technique; (**a**) original s-X-ray detected image, (**b**) image result after taking the median filtering technique.

Even though, the CMOS sensor were protected by the aluminum box, salt and pepper noise is always presented in the image due to the subjecting of electronics to X-ray. Thus, the median filtering technique was employed to completely remove this noise from the image as shown in Figure 3.

#### 2.1.3. Image Registration

**Figure 4:**
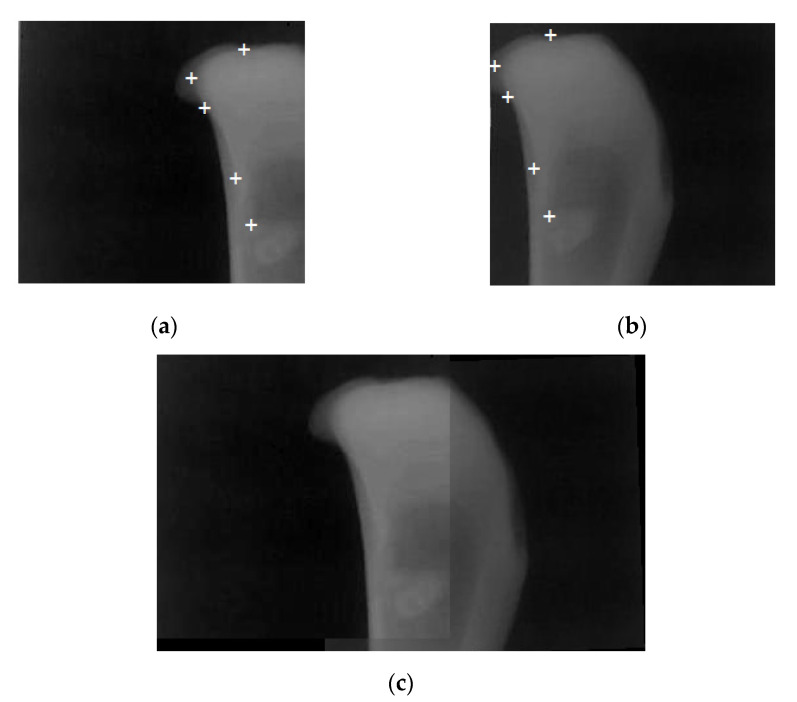
Image registration. (**a**) Image captured from right CMOS sensor; (**b**) image captured from left CMOS sensor; (**c**) aligned image. White crosses are the matched point.

To increase resolution of our indirect X-ray detector, multiple CMOS sensors can be used to capture the intensifying screen image. For system of two CMOS sensors, the two CMOS sensors after perfectly aligned was positioned to capture some parts of the complete image. The common part of the complete image captured by adjacent CMOS sensor is need in the registration process. This section will describe the basic concept of image registration process. To find small parts of an image which match a template image, template matching is used. The basic method is to loop through all the pixels in the search image and compare them to the pattern. Figure 4 shows the template matching technique which taken in this research. The process automatically selected highly detailed and unique templates from one image and located the templates in another image of the same scene. After establishing the correspondences using the template matching, the set of corresponding matching points of the two images are then used to compute the transformation matrix T which is consequently used to align the two images. When there is an absence of non-linear transformation, an affine transformation between the two to-be-aligned images is modeled as,

(3) Y=T⋅X

where X and Y are the matrices of matched points between the two images and,

$$X=\left[\begin{array}{cccc} x_{1} & x_{2} & \cdots & x_{n}\\ y_{1} & y_{2} & \cdots & y_{n}\\ 1 & 1 & \cdots & 1 \end{array}\right]$$

$$Y=\left[\begin{array}{cccc} u_{1} & v_{2} & \cdots & u_{n}\\ u_{1} & v_{2} & \cdots & v_{n}\\ 1 & 1 & \cdots & 1 \end{array}\right]$$

where *n* is the matched minutiae points between the two images, and T is the affine transformation matrix,

$$T=\left[\begin{array}{ccc} a_{11} & a_{12} & a_{13}\\ a_{21} & a_{22} & a_{23}\\ 0 & 0 & 1 \end{array}\right]$$

Similarly, T can be estimated using least squre error using,

(4) $$T={\left(X^{T}X\right)}^{-1}(X^{T}Y)$$

After T is estimated, the two images captured from the same intensifying screen can then be aligned. The result of image registration is shown in Figure 4. 

#### 2.1.4. Image Stitching

**Figure 5:**
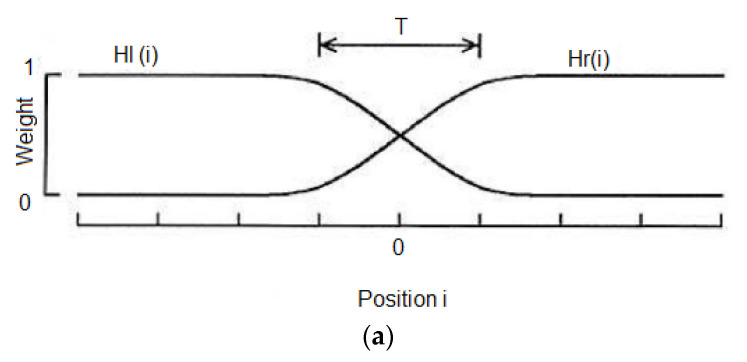

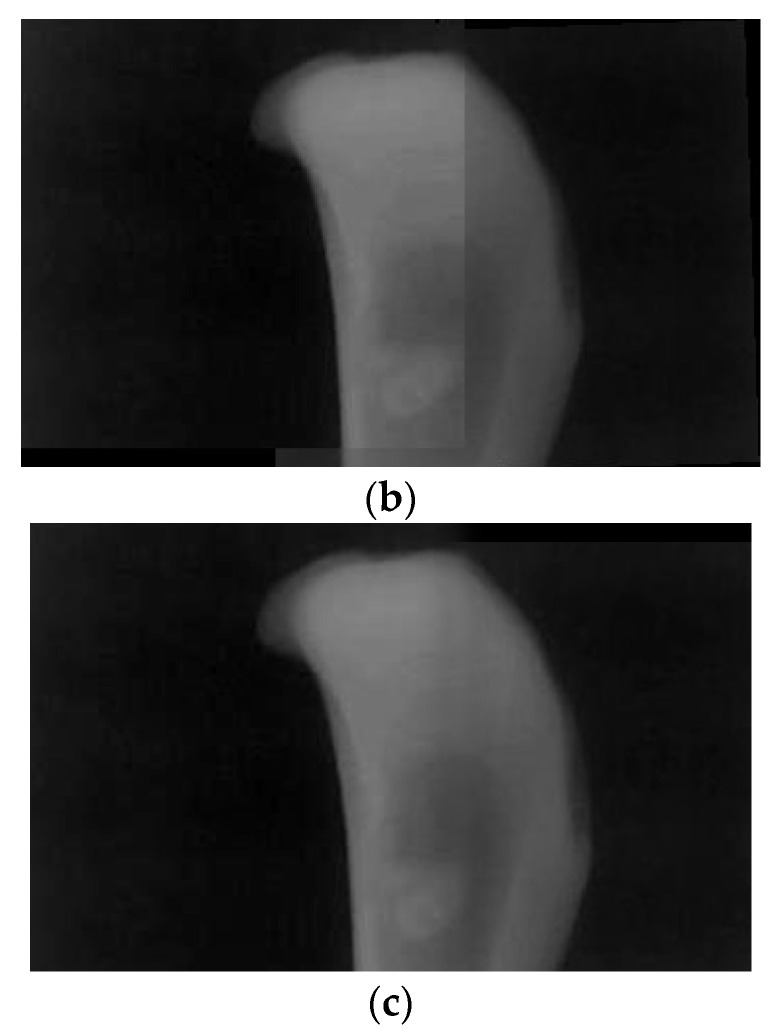
Image stitching; (**a**) Weight function; (**b**) Before image stitching (**c**) Results of image stitching.

The result from the differences of gray level is frequently unavoidable since such factors are different in sensor position. Thus, weighted average technique is required to modify image gray scales in the vicinity of a boundary to obtain a smooth transition between overlapped images. Each image is multiplied by a weighting function which decreases monotonically across its border; the resulting images are then summed to form the mosaic. Example weighting functions are sigmoid function as shown in Figure 5a. The width of the transition zone **T** is a critical parameter for this method. Figure 5b,c shows the images stitched by the weighted average technique.

### 2.2. Modified Beer-Lambert Law for Bone Mineral Density Estimation

Bone mineral density using dual energy X-ray is based on the well-known Beer-Lambert law. When a tissue consisting of bone and soft tissue is radiated with X-ray, intensity of transmitted X-ray *I* related to incident X-ray $I_{0}$ can be described as a modified Beer-Lambert law Equation,

(5) $$I=I_{0}e-\left(\mu_{s}\rho_{s}+\mu b\rho bXb\right)$$

where $\mu_{s}$ is mass attenuation coefficient of soft tissue (cm/g), $\mu_{b}$ is mass attenuation coefficient of bone (cm/g), $\rho_{b}$ is density bone (g/${\mathrm{cm}}^{3})$, $\rho_{s}$ is density of soft tissue (g/${\mathrm{cm}}^{3})$, *X_s_* is thickness of soft tissue (cm) and *X_b_* is thickness of bone (cm). Equation can be linearized as Equation (6),

(6) $$\ln\left(\frac{I_{0}}{I}\right)=(\mu_{s}\rho_{s}X_{s}+\mu_{b}\rho_{b}X_{b})$$

As taking X-ray is the projection of three dimension to two dimension, in Equation (6), $\rho_{b}X_{b}$ bone density in g/cm^2^ and $\rho_{s}X_{s}$ soft tissue density in g/cm^2^ are the unknown to be solved. The other terms can be considered as the constant parameters. To solve for the unknown, two energy levels, high and low level, are used to radiate the tissue. Then the following equations can be derived for low and high energy level of X-ray, respectively.

(7) $$\ln{\left(\frac{I_{0}}{I}\right)}^{l}=(\mu_{s}\rho_{s}X_{s}+\mu_{b}\rho_{b}X_{b})$$

(8) $$\ln{\left(\frac{I_{0}}{I}\right)}^{h}=(\mu_{s}\rho_{s}X_{s}+\mu_{b}\rho_{b}X_{b})$$

Define *k* as the ratio of absorption coefficient of soft tissue at high energy level and low energy level.

(9) $$k=\frac{\mu_{s}^{l}}{\mu_{s}^{h}}$$

Multiply *k* to Equation (8) and subtract the result from Equation (7), we derive,

(10) $$\ln{\left(\frac{I_{0}}{I}\right)}^{l}-k\ln{\left(\frac{I_{0}}{I}\right)}^{h}=(\mu_{b}^{l}\rho_{b}X_{b}+k\mu_{b}^{h}\rho_{b}X_{b})$$

Rearrange Equation (10) to derive,

(11) $$\frac{\ln{\left(\frac{I_{0}}{I}\right)}^{l}-k\ln{\left(\frac{I_{0}}{I}\right)}^{h}}{\mu_{b}^{l}-k\mu_{b}^{h}}=\rho_{b}X_{b}$$

The $\rho_{b}X_{b}\;$ is bone mineral density in g/cm^2^.

(12) $$BMD=\frac{\ln{\left(\frac{I_{0}}{I}\right)}^{l}-k\ln{\left(\frac{I_{0}}{I}\right)}^{h}}{\mu_{b}^{l}-k\mu_{b}^{h}}$$

Bone mineral content can be computed from,

BMC = BMD × Area (13)

**Figure 6:**
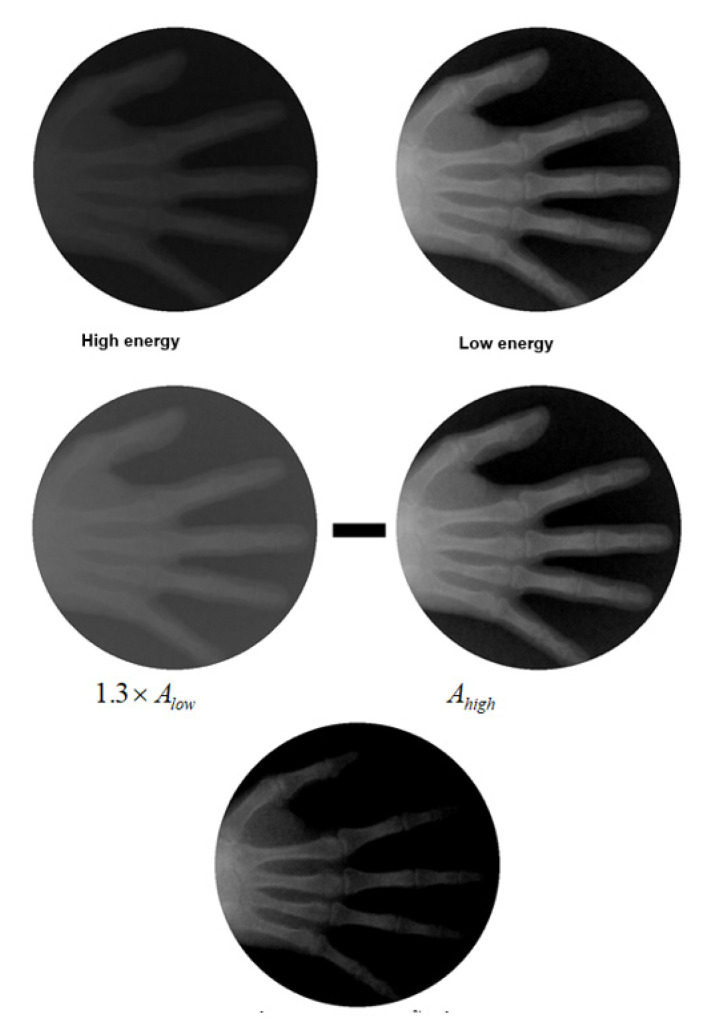
Process of determining *k*. By subtracting the high-level detector image from *k* multiplies the low-level detector image, *k* is adjusted until no soft tissue appeared on the subtracted image (the bottom).

In practice, $I_{0}$ can be derived from detector image when taking X-ray with no object. Factor *k* is determined by subtracting the high-energy level detector image from a *k* time the low-energy level detector image. *k* is fine-tuned until no soft tissue appeared on the subtracted image. Figure 6 shown the process of determining *k*.

### 2.3. Area Determination

The proposed bone mineral content using dual energy X-ray is applied to middle phalanges of the middle finger. To compute mineral content, area of the phalanges bone is needed. To find the area, active contour [25] is implemented. 

A parametric active contour or snake is a curve, with parameter s [0,1]. The curve can move on the image plane under the influence of two types of forces-internal and the external forces. The former constrains the snake to be smooth while the latter guides the snake to seek desirable image properties, such as edges. The external forces are computed from the image data. Such an active contour model seeks to minimize the following functional [11],

(14) $$E_{Snake}=\int_{0}^{1}\frac{1}{2}\cdot [\alpha (s)\cdot |\nu_{s}(s){|}^{2}+\beta (s)\cdot |\nu_{ss}(s){|}^{2}]+E_{Ext}ds$$

where terms in the bracket associated with internal energy and $E_{Ext}$ is external energy. The $v_{s}$ (s) and $v_{ss}$ (s) of the energy control the smoothness and the rigidity of the contour, respectively, by exercising on the parameter α and β, respectively. In order to attract snake contour to salient features in images, the external energy is needed. The typical external energy designed to lead an active contour toward object boundaries are,

(15) $$E_{ext}^{1}=-|\nabla I(x,y){|}^{2}$$

(16) $$E_{ext}^{2}=-{\left|\nabla [G_{\sigma }(x,y)*I(x,y)]\right|}^{2}$$

where *I (x, y)* is a gray-level image, $G_{\sigma }\left(x,y\right)$ is a two-dimensional Gaussian function with standard deviation σ and ∇ is the gradient operator. The key problem of a traditional external force is its limited capture range. Increasing σ can enlarge the capture range but the larger will result in inaccurate boundary localization. Several methods such as distance potential force, gradient flow vector force [26] has been proposed to significantly increase the capture range of a traditional snake. However, they all use only edge information. Gradient flow vector force is derived by the following energy function,

(17) $$\begin{array}{l} E_{GVF}(u,v)=\frac{1}{2}\iint g\left(\left|\nabla f\right|\right)\left(u_{x}^{2}+u_{y}^{2}+v_{x}^{2}+v_{y}^{2}\right)dxdy\\ +\frac{1}{2}\iint \left(1-g\left(\left|\nabla f\right|\right)\right)\left({\left(u-f_{x}\right)}^{2}+{\left(v-f_{y}\right)}^{2}\right)dxdy \end{array}$$

where *f(x*) is the edge map and *g* is a decreasing function of the gradient magnitude defined as,

$$f(x,y)=-{\left|\nabla [G_{\sigma }(x,y)*I(x,y)]\right|}^{2}$$

$$g\left(\nabla f\right)=exp\left(-\left(\frac{\left|\nabla f\right|}{k}\right)\right)$$

**Figure 7:**
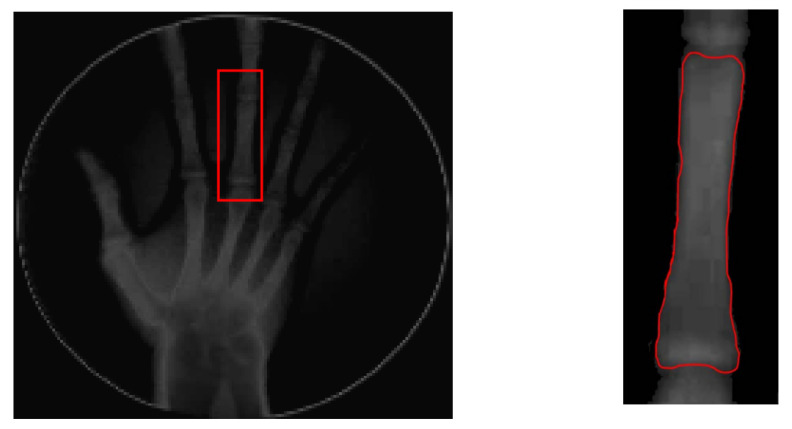
Active contour used to find the contour of middle phalanges of middle finger.

To implement GVF active contour, the initial contour, mostly circle, is placed inside the object. Force of GVF computed from edge image is then iteratively drive the initial contour to the edge contour. Figure 7 shows result of active contour to find the contour of middle phalanges of middle finger. The detected contour is then used to compute the area of middle phalanges of middle finger by summing all pixels confined within the detected contour.

### 2.4. Procedure of Bone Mineral Density Determination

The process to determine bone mineral density can be divided into 2 processes, i.e., (i) preprocess and (ii) measurement process. Preprocess can be carried out in daily basis while measurement process must do every time when bone mineral density determination is needed. The preprocess is used to derive the detector image of incident X-ray $I_{0}$ where the measurement process is used to compute bone mineral density and bone mineral content.

(i) Preprocess for Bone Mineral Density Determination

Step (i)Turn on X-ray and set tube current at 25 mAStep (ii)Set tube voltage at high kVp. With no object, start X-ray exposure for 40 ms. X-ray detector captures and saves captured image as $I_{0}^{h}$. Synchronized trigger algorithm is used to automatic capture the image by monitor the average intensity of the detected image. When the X-ray is exposed on the detector, the averaged intensity increased. By setting the threshold, when the averaged intensity is more than the threshold, image on the intensity will be captured and saved.Step (iii)
Set tube voltage at low kVp. With no object, start X-ray exposure for 40 ms. X-ray detector captures and saves captured image as $I_{0}^{l}$

(ii) Measurement Process for Bone Mineral Density Determination

Step (i)Turn on X-ray and set tube current at 25 mAStep (ii)Set tube voltage at high kVp. With object, start X-ray exposure for 40 ms. X-ray detector captures and saves captured image as high-energy level detector image $I^{h}$. Delay for 2 s. Step (iii)Set tube voltage at low kVp. Start X-ray exposure for 40 ms. X-ray detector captures and saves captured image as low-energy level detector image, $I^{l}$. Delay for 2 sStep (iv)Compute
$\ln{\left(\frac{I_{0}}{I}\right)}^{l}\;$ and
$\ln{\left(\frac{I_{0}}{I}\right)}^{h}$
Step (v)Compute bone mineral density Equation (12)Step (vi)Compute area using GVF active contour and bone mineral content Equation (13)

## 3. Experiments and Results

We conducted experiments into 2 categories including X-ray detector calibration experiment and bone mineral density experiment. The objective of X-ray detector calibration experiment is to seek the optimal exposure setting for dual exergy X-ray and to test the characteristics of X-ray detector. The objective of bone mineral density experiment is to test the performance of bone mineral density system both on calcium phantom and on human subject. 

### 3.1. X-ray Detector Calibration

**Figure 8:**
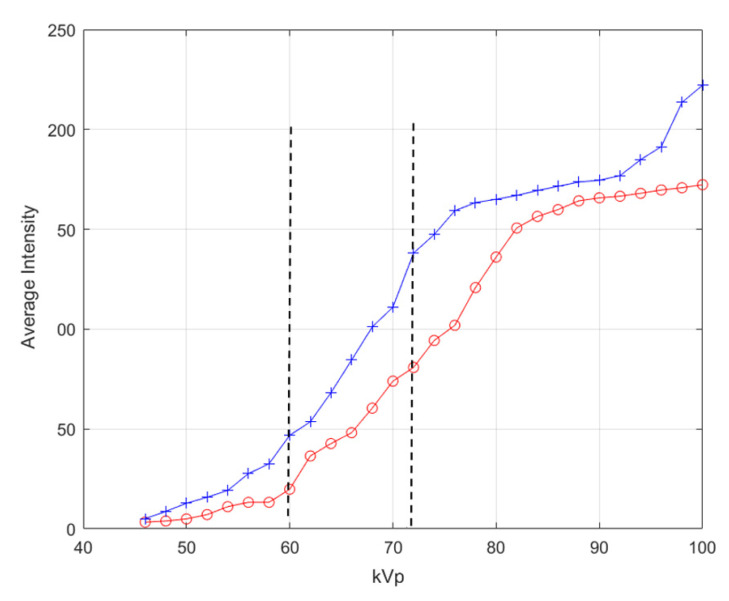
Linearity test of indirect X-ray detector; Between dash line shows the linear response. (red: 25 mA, blue: 50 mA).

To test the reliability of the indirect X-ray detector, we perform linearity test, resolution test and repeatability test. Linearity test is to establish the range of tube voltage setting for each tube current setting. The dedicated microcontroller X-ray apparatus can set the tube voltage from 40–100 kVp with an increment of 2 kVp per step. Two tube current can be set which are 25 mA and 50 mA. To perform linearity test, tube volage is set from 46 kVp to 100 kVp for each tube current setting. With no object, X-ray exposure is activated while the X-ray detector captures the image of intensifying screen. Synchonized trigger is used to capture image automatically. Averaged intensity for each setting are plotted against the kVp setting. The result is depicted in Figure 8. We can conclude from the graph in Figure 8 that the indirect X-ray detector demonstrated liner response between 60–72 kVp for both 25 mA and 50 mA tude current. We hence opt to set the X-ray exposure for low energy level and high-energy level at 60 kVp, 25 mA and 72 kVp 25 mA, respectively.

The next experiment for X-ray detector calibration is repeatabilty test. To ensure the robustness of our detector, we test the X-ray detector at the same setting for high and low energy level for 10 times. The standard deviation of the averaged intensity is computed. Result is shown in Table 2.

**Figure 9:**
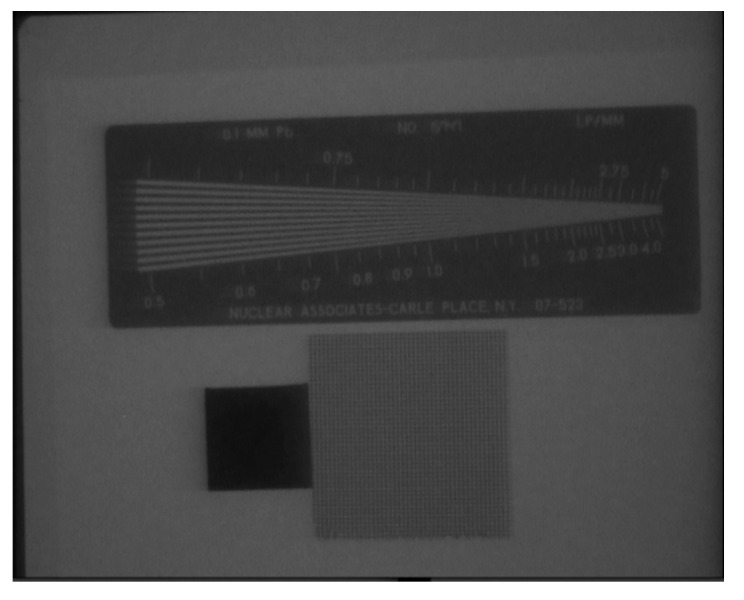
Resolution test of X-ray dectetor. 1.2 lp/mm is observed.

Resolution test is also performed on the X-ray detector. Many factors can affect the resolution of the detector including focal length (filament size) of the X-ray tube, type of image intensifying screen, resoultion of detector, i.e., the CMOS sensor. We have tested resolution of our X-ray detector using flouroscopic quality control test tool model 07-637. The results in Figure 9 indicated that our detector resultion is about 1.2 lp/mm. 

### 3.2. Perfomance Test on Calcium Phantom

**Figure 10:**
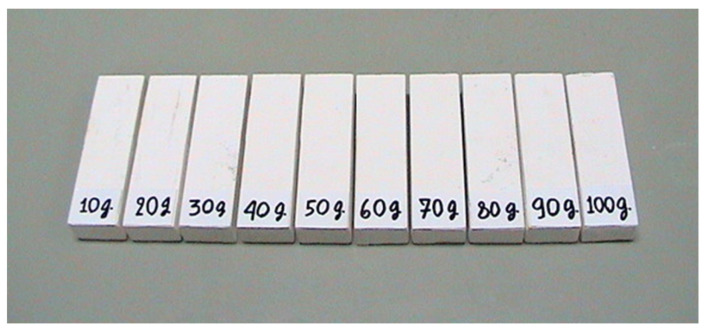
Calcium phantom.

**Figure 11:**
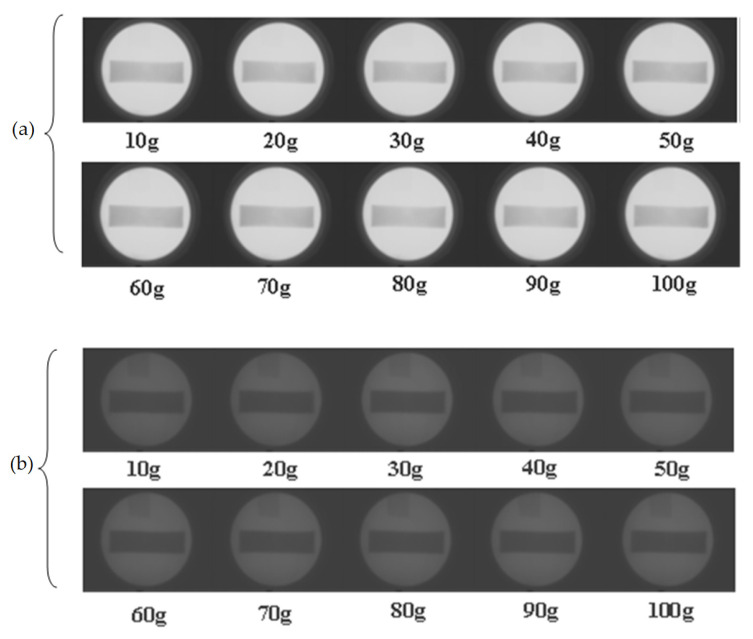
High (**a**) and low (**b**) energy detector image of calcium phantom.

**Figure 12:**
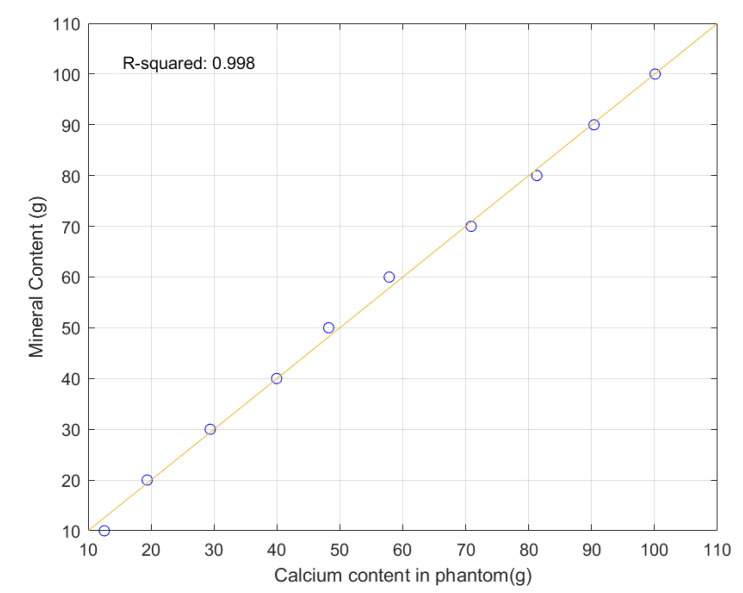
Plot of real calicium content (*x* axis) against computed mineral (calcium) content (*y* axis).

Prior to test the proposed bone minereral density measuremenst system with human subject, we have tested the system with calcium phantom. The calcium phatom is created by mixing 40 g of plaster power, 80 mL of water and vairious amount of calcium powder starting from 10 g to 100 g with an increment of 10 g. The dimension of calcium phantom is 4 cm × 14 cm × 0.8 cm (W × L × H) and hence the top surface area of the phantom is 56 cm^2^. Figure 10 shows the 10 calcium phantoms. Figure 11 shows the high and low energy level detector image of calcium phantom. Result of mineral density and mineral content is tabulated in Table 3. Graph of computed mineral content using the proposed system and the real content of calcium is depiced in Figure 12 with R^2^ of 0.998. 

### 3.3. Performance Test on Subject

**Figure 13:**
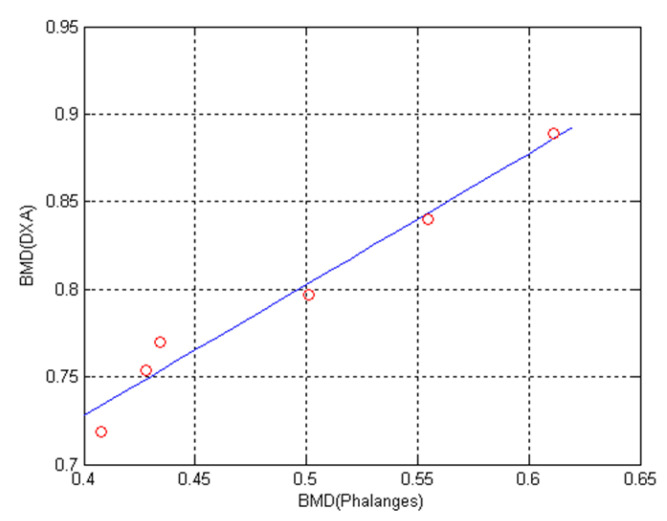
Graph between BMD computed from the proposed bone mineral density system against that measured by commercial bone mineral density system (R^2^ = 0.969) Equation of fitting line y = 0.4285 + 0.7482x.

**Figure 14:**
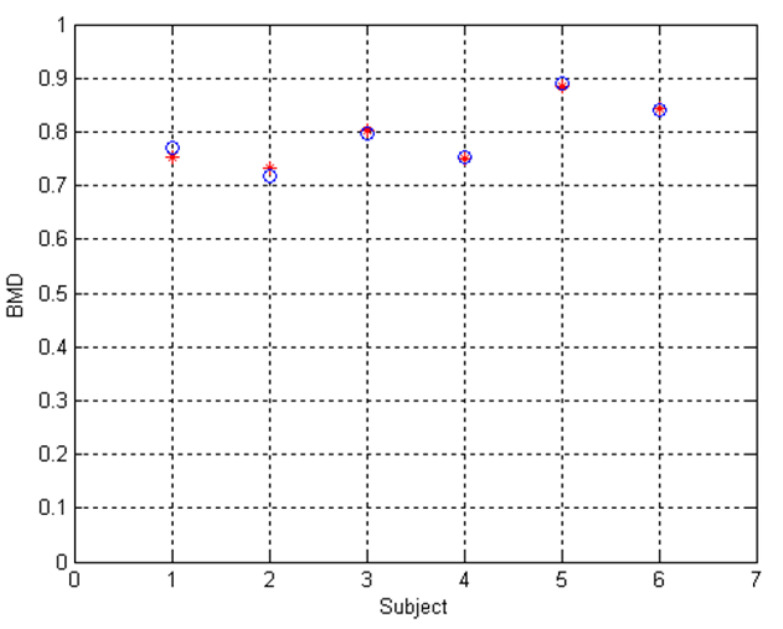
Plot of BMD computed from the proposed bone mineral density system (+) with measured by commercial bone mineral density system (o) after normalization.

The proposed bone-mineral density measurement system has been tested with 6 male subjects with age between 25–29 years. The measurement is compared with commercial bone-mineral density measurement apparatus model DSC-600EX ALOKA which is also based on dual-energy X-ray. The results of bone mineral density and bone mineral content using our proposed system is shown in Table 4. The comparison of BMD measurement of our system and the commercial model is shown in Table 5 and Figure 13. From Figure 13, the relation between BMD measurement of our system and the commercial system is linear with R^2^ equals 0.969. The BMD measurement of our system can be normalized to the BMD value of the commercaial model using linear equation y = 0.4285 + 0.7482x. The normalized BMD value is plotted against the BMD value of the commercaial model as shown in Figure 14. The mean square error between the normalized BMD value and that of the commercaial model is 0.0000981.

### 3.4. Design for Bone-Mineral Density Measurement

**Figure 15:**
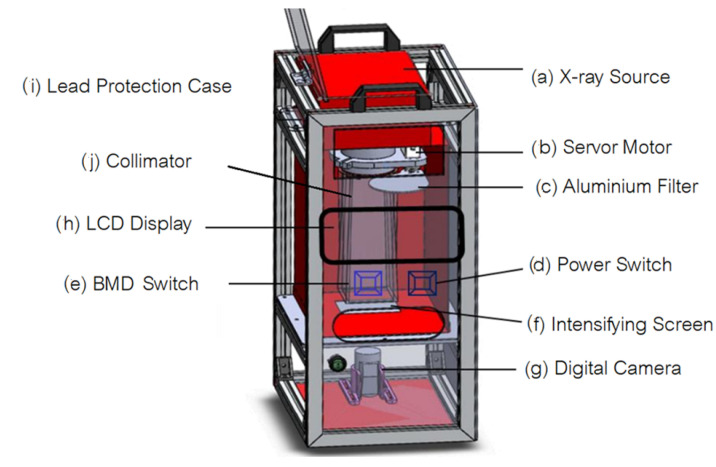
Portable Bone-Mineral Density Measurement System.

To provide mobility of our system, we have designed a portable bone-mineral density measurement system based on dual energy X-ray proposed by this manuscript as shown in Figure 15. Installed in the lead-protection case (i), the main components of the system are X-ray source (a) and x-detector (j, g). The X-ray source is a handheld dental X-ray device which is mounted on the top of the case. Our indirect X-ray detector consists of intensifying screen and CMOS Sensor. Intensifying screen converts X-ray to visible light. An image of illuminated intensifying screen is then captured with digital for further processing. To control energy level of X-ray, a 1-mm aluminum filter is used. The aluminum filter (c) is controlled by servo motor (b). When lower-energy X-ray is needed, servo motor will move the aluminum filter into the collimator (j) to attenuate the energy of X-ray. Raspberry Pi microcontroller is used to control the operation of the system including image acquisition and image processing. 

## 4. Discussion

This research concerns the design and construction of low-cost bone mineral density measurement system based on dual energy X-ray. X-ray source used in our research is a dedicated microcontroller X-ray apparatus with is communicated to personal computer using serial communication. X-ray detector is key contribution of this research. An indirect X-ray detector is designed using one or multiple CMOS sensors capturing image on the intensifying screen and transferring the image to personal computer via USB port for further analysis. Despite the promising result when compared with the measurement from the commercial model, there are a number of issues that are needed to be discussed for the completeness of the manuscript.

(i) When compared the results of our bone mineral density measurement system with the measurements from the commercial model, our BMD seems to be underestimated. There are factors that may cause the underestimation. One of the factors is the error of the setting kVp X-ray tube voltage from the true value of tube voltage. To compute bone mineral density using modified Beer Lambert law, mass attenuation coefficient of bone and soft tissue are needed. The mass attenuation coefficient is a function of X-ray energy which depends on the kVp supplied to the X-ray tube. Any error of tube voltage could directly result the error in bone mineral density measurement. The other factors may cause by the low sensitivity of our indirect X-ray detector upon compared with the X-ray detector used in the commercial mode which is more sensitive. Despite the measurement error, however, the error related to hardware is consistent and hence compensable through calibration.
(ii) Our proposed bone mineral density system aims to be used for screening process at district hospital. The measurement results may inform patient with low risk, moderate risk, and high risk of bone mineral deficiency. For those with medium to high risk will be recommended to have further diagnosis with commercial model of bone mineral density measurement at the center hospital. Our system does not intend to use for osteoporosis diagnosis as the diagnosis requires statistical analysis of bone mineral density data from a large population.
(iii) The effective dose of the subject using the proposed bone mineral density system is equivalent to two times the effective dose of extremity radiograph which is about 2 μSv [27]. In modern dual exergy X-ray bon scanner, the effective does varies from 1 μSv to about 15 μSv for fan beam and about 18 μSv for cone beam model [28]. The effective does of the proposed system is hence less than the commercial model. Moreover, in the designed portable model, X-ray source is installed in the lead-shield case and the collimator is used to direct the X-ray only on the finger area. As a result, the real effective dose is less than the predicted value.
(iv) The area determination is based on active contour and hence requires the placement of initial contour. The initial contour which is a circular contour with diameter 50 pixel must be placed inside and at the center of the middle phalanges of the middle finger. In the real experiment, the subject hand must be fixed in position so that the X-ray central ray is at the center of the middle phalanges. To convert pixel unit to physical unit, image of a known dimension of square metal object exposed with X-ray is captured with the indirect X-ray detector.
(v) The proposed indirect X-ray detector is the main contribution of the research. The cost of construction is very low when compared with commercial detector. This make to overall cost of the proposed bone mineral density system is almost 10 times cheaper than the commercial model. There are two issues of X-ray detector are needed to be addressed. First, our detector used CMOS sensor. Modern CMOS sensor can achieve up to 12 Mega pixels. This makes the proposed X-ray detector upgradable with reasonable cost. Furthermore, by concatenating more CMOS sensors, more pixels covering the same active area, the resolution can be increased. Second, the intensifying screen used in the X-ray detector also affects the performance. Normally, there are two types of intensifying screen which are high resolution and high sensitivity type. For high resolution type, the X-ray phosphor grain size is smaller than that of the sensitive one. However, the resolution type is less sensitive. In our research, we opt to use the sensitive screen by considering the fact that the highly sensitive type requires low effective dose than the high resolution. The efficiency of our X-ray detector however is not as good as the commercial one. The light emitted from Gd_2_O_2_S: Tb intensifying screen is 545 nm. The spectral response of the CMOS sensor is not specific at any wavelength but the whole visible light. The efficiency is hence not as good as the commercial where the response of the emitted light and detector is perfectly matched. To improve efficiency, high-sensitive CMOS sensor can be used.
(vi) One the important factor in determining dual energy level for DEXA is k-edge effect. At the k edge, X-ray energy drops rapidly due to the photoelectric phenomenon. To avoid k-edge effect, many techniques can be used including using X-ray detector with k-edge filter or setting the two-operating point above the k-edge energy level. The k-edge of calcium in the bone which is considered as high atomic number is about 4 kev. In general, the k-edge of bone is below conventional diagnostic energies, the tail of k-edge does fall into diagnostic energies. As a result, bone absorbs much more of the lower energy X-rays than soft tissues do. Our proposed dual energy X-ray absorption apparatus set the high and low X-ray energy level at 40 kVp and 72 kVp, respectively. The average energy is approximate 1/3 of kVp. The average kev for high and low X-ray energy level are 13 kev and 24 kev, respectively, which is above the k-edge of calcium.

## 5. Conclusions

This research concerns the design and construction of a low-cost bone mineral density and content based on dual energy X-ray absorption or DEXA. The system consists of two main components: (i) X-ray controller and (ii) X-ray detector. X-ray controller is the dedicated microcontroller 50-mA X-ray apparatus that is interfaced with personal computer via serial communication. Our X-ray detector is designed by using a CMOS sensor capturing image from conventional X-ray intensifying screen. The captured image is transmitted to personal computer for further processing. Bone mineral density system is controlled by inhouse software installed on the personal computer. The inhouse software will control sequence of bone mineral density and content measurement process and the post processing related to digital image processing that uses OpenCV. Bone mineral density and content will measure on the phalanges of the middle finger. To derive bone mineral content, area of phalanges is needed. We exploit the principle of GVF active contour for area computation. The initial circular contour is placed in the middle of phalanges. The GVF external force compute from edge image is then iteratively drive the initial contour to fit the phalanges contour. A number of tests have been performed on the X-ray detector including linearity, repeatability and resolution test with satisfactory result. Performance tests have been conducted on the calcium phantom with known calcium content and on human subjects. Compared with the measurement derived from the commercial DUXA, after being calibrated our bone mineral density measurement system can provide the measurement close to that of the commercial with mean-squared difference of 0.00981%. 

## Figures and Tables

**Table 1:** Key parameter of CMOS Sensor.

Parameter		Typical Value
Optical format		1/2 inch (5:4)
Actual image size		6.66 mm (H) × 5.32 mm (V)
Actual pixel size		1280 H × 1024 V
Shutter type		Electronic rolling shutter (ERS)
Maximum data rate/Master clock		48 MPS/48 MHz
Frame rate	SXGA (1280 × 1024)	30 fps for progressive scan programmable
ADC resolution		10-bit, on chip
Responsively		2.1 V/lux-sec
Dynamic range		68.2 dB
SNR max		45 dB

**Table 2:** Repeatability test of indirect X-ray detector.

No	Averaged Intensity	
	60 kV	76 kV
1	13.525	31.252
2	13.558	31.097
3	13.488	31.224
4	13.555	30.909
5	13.464	31.343
6	13.482	31.206
7	13.516	31.196
8	13.45	31.302
9	13.489	30.914
10	13.508	31.143
Mean ± STD	13.503 ± 0.035	31.158 ± 0.148

**Table 3:** Mineral density and concentration of calcium phantom.

Ingredient: Plaster Power/Water/Calcium	BMD	BMC
(g/cc/g)	g/cm	(g)
40/80/10	0.2245	12.5727
40/80/20	0.3461	19.3847
40/80/30	0.5249	29.3996
40/80/40	0.7134	39.9538
40/80/50	0.8611	48.2229
40/80/60	1.0328	57.8416
40/80/70	1.2653	70.8574
40/80/80	1.4516	81.294
40/80/90	1.6139	90.3785
40/80/100	1.7873	100.094

**Table 4:** Result of bone mineral density and bone mineral content in 6 subjects.

Subject No	Detector Image	BMD (g/cm^2^)	BMC (g)
1	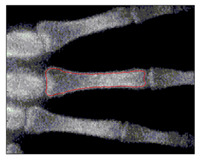	0.434	7.443
2	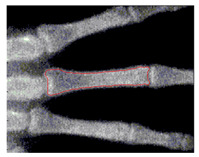	0.4077	9.07
3	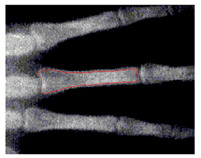	0.5013	8.747
4	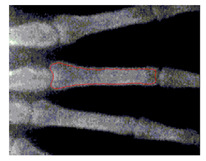	0.428	6.817
5	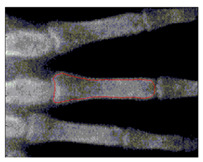	0.6113	7.072
6	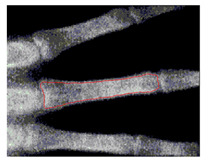	0.5548	8.476

**Table 5:** Comparison between the BMD measured from proposed bone mineral density system and that measurd from commercial bone mineral density system.

Subject	Measured BMD	BMD Commercial
1	0.434	0.77
2	0.4077	0.719
3	0.5013	0.797
4	0.428	0.754
5	0.6113	0.889
6	0.5548	0.84
